# Bulimic symptoms in a sample of college women: disentangling the roles of body size, body shame and negative urgency

**DOI:** 10.1007/s40519-019-00771-z

**Published:** 2019-09-25

**Authors:** Simon E. Dalley, Glenda G. Bron, Iona F. A. Hagl, Frederic Heseding, Sabine Hoppe, Lotte Wit

**Affiliations:** Rijksuniversiteit Groningen, Grote Kruisstraat 2/1, Groningen, The Netherlands

**Keywords:** Body size, Body shame, Impulsivity, Negative urgency, Bulimic symptomatology

## Abstract

**Abstract:**

Purpose This study set out to disentangle the roles of body size, body shame and negative urgency on bulimic symptomatology in a sample of college women. We predicted that body shame would mediate the relationship between body size and bulimic symptomatology: with increasing body size, the greater would be the experience of body shame and, in turn, the greater the bulimic symptomatology. We also predicted that negative urgency would exacerbate this mediation pathway, and that the moderated mediation model would occur over and above current levels of depression.

**Method:**

A convenience sample of 237 college women indicated their age, height and weight and then completed measures of body shame, negative urgency, depression and bulimic symptomatology. Bootstrap analysis was used to test the predicted moderation mediation model.

**Results:**

The bootstrap analysis supported all predictions. Thus, with greater the increase in body size, the greater was the body shame and the more frequent bulimic symptomatology. Furthermore, negative urgency moderated the relationship between body shame and bulimic symptomatology, such that those with both higher negative urgency and body shame had more frequent bulimic symptomatology.

**Conclusions:**

Results suggest that those college women higher in both BMI and negative urgency are likely to experience higher levels of bulimic symptoms. These women may benefit from emotion regulation interventions targeted at preventing, as well as coping effectively with, the experience of body shame.

**Level of evidence:**

V: cross-sectional descriptive study.

## Introduction

The binging and purging behaviors that characterize bulimia nervosa are highly prevalent on college campuses [1]. Unfortunately, bulimic symptomatology can have a significant negative impact on a student’s physical and psychological well-being, as well as on their academic performance [2, 3]. Since body shame is linked with bulimic symptoms [4–7], the aim of this paper is to elucidate a process by which body shame can influence the expression of those symptoms, and by so doing identify those individuals who are most at risk of manifesting bulimic pathology. Specifically, we propose that the extent of the bulimic symptoms arising from body shame occurs in the context of an increasing body size, such that body shame mediates the relationship between body size and bulimic symptomatology. We also propose that negative urgency, an individual difference variable associated with the maladaptive regulation of negative emotions [8], exacerbates the relationship between body shame and bulimic symptomatology over and above general negative affect.

Shame is thought to arise as a result of a failure to live up to a personally significant internalized standard and is characterized by self-criticism, feelings of worthlessness and inferiority [9–12]. Such being the case, shame about the size of one’s body could be said to be an emotion that is experienced by a significant number of women in contemporary Western culture [6]. In this regard, research suggests that because of pressure from the media, peers and family, many women in affluent Western cultures have internalized a cultural body standard that is characterized by an extreme level of thinness [13, 14]. Unfortunately, because this internalized standard is almost impossible to achieve, many women experience significant negative affect about their body size [14, 15]. Indeed, presumably because greater body size is an indicator of distance from the internalized thin body standard, increasing body size has been found to be the most consistent correlate of body dissatisfaction in women [16]. With this in mind, we reason that with increasing body size, the greater will be the experience of body shame. Support for our reasoning comes, firstly, from general emotion theory, whereby shame is thought to arise in response to a perception that one has failed to live up to an internalized ideal [10]. Secondly, and more specifically, support also comes from body image research indicating that greater thin ideal internalization in women is associated with greater body shame [15, 17, 18].

Within the body image domain, body shame has been found to be associated with bulimic symptoms in both clinical and non-clinical samples [19–22]. One explanation for the association between body shame and bulimic symptomatology is the escape theory of binge eating [6, 23]. Central to escape theory is the idea that because body shame is an exceptionally unpleasant emotion, people are motivated to escape from such an experience by engaging in the binging and purging behaviors characteristic of bulimia nervosa [6, 24]. Accordingly, we therefore expect that body shame will mediate the relationship between body size and bulimic symptoms: with increasing body size, the greater will be the experience of body shame and, following on from this, the greater the bulimic symptomatology. However, we also expect that this mediation pathway will be stronger for some women than others.

Impulsivity is generally characterized by a tendency to act in a rash or spontaneous manner [25]. The construct of impulsivity has long been linked to the binging and purging components of bulimia nervosa [26]. For example, Waxman [27] reported that among individuals diagnosed with an eating disorder, those who engaged in purging and behaviors also had the highest levels of trait impulsivity. Furthermore, and of particular relevance for this study, Higgins and colleagues [6] recently reported that impulsivity moderated the relationship between body shame and bulimic symptoms in a sample of young black women. Yet despite these relationships, impulsivity is a broad construct consisting of different components [25], and so there is a need to clarify and pinpoint which component is the most relevant for bulimic symptomatology [28]. One component of impulsivity, which appears to be particularly relevant for both binging and purging, is negative urgency: the tendency to act rashly, specifically when faced with negative emotion. For example, negative urgency is the most predictive component of impulsivity for binge eating and purging behaviors, and has also been shown to be a prospective risk factor for the development of bulimic symptoms [28–30]. From an escape theory perspective, and given the nature of negative urgency, one would expect that those higher in negative urgency to be especially likely to engage in impulsive escape behaviors in the context of increasing body shame [8]. We therefore expect that with increasing body shame, and the higher the level of negative urgency, the greater will be the propensity to engage in rash behaviors in the form of bulimic symptomatology.

Finally, we recognize that the presence of other negative emotions can also play a role in the predicted model. For example, the experience of depression has also been found to predict bulimic symptoms [31, 32]. It could also be the case therefore that bulimic symptomatology is also a way to escape the unpleasant feelings of depression. Thus, to clarify and understand the unique role of body shame in the predicted model, we controlled for current depressive symptoms.

**Fig. 1:**
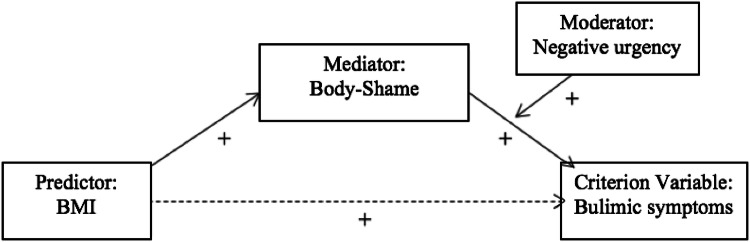
Proposed moderated-mediation model

To summarize, in a sample of college women, we predict a model in which body shame mediates the relationship between body size and bulimic symptomatology, and that negative urgency  moderates the relationship between body shame and bulimic symptomatology (see Fig. 1). We also predict that the significant effects of this moderated mediation model will occur over and above the influence of current depressive symptoms.

## Method

### Participants

Two hundred and thirty-seven female students originally volunteered to take part in this cross-sectional study. The participants were recruited as part of a convenience sample at the university library. Twenty-eight participants were excluded from the study because of incomplete questionnaires, leaving a total of 209 participants for statistical analysis. Ages ranged from 18 to 30 years, with a mean age of 21.59 (SD = 3.17).

### Materials

#### Body size

Body size was operationalized by calculating body mass index (BMI). Specifically, each participant’s BMI was calculated from self-reported height and weight. Previous studies have shown a variation by 1–3.5% between actual height and weight and subjectively reported values [33].

#### Negative urgency

The four-item negative urgency subscale of the short English version of the Urgency, Premeditation (lack of), Perseverance (lack of), Sensation Seeking, Positive Urgency, Impulsive Behavior Scale (UPPS-P) was used to measure the participant’s level of negative urgency [34]. This subscale consists of four items (e.g., “When I am upset I often act without thinking” and “When I feel bad, I will often do things I later regret in order to make myself feel better now”) with anchors ranging from 1 (strongly disagree) to 4 (strongly agree). The subscale has previously been shown to have adequate internal consistency (*α *= 0.78) in a sample of undergraduate students. In the current sample, the Cronbach’s alpha was 0.77.

#### Body shame

The body shame subscale of the objectified Body Consciousness Scale was used to measure the level of body shame of the individual participants [35]. This subscale consists of eight items (e.g., “I would be ashamed for people to know what I really weigh” and “I feel like I must be a bad person when I don’t look as good as I could”). Participants were instructed to respond to these items on a seven-point Likert scale ranging from 1 (strongly disagree) to 7 (strongly agree). Higher total scores on this subscale indicate that the individual experiences higher levels of body shame. Its psychometric properties have previously been found to include a satisfactory internal consistency (*α* = 0.75) in a sample of undergraduate women [35]. In the current sample, the Cronbach’s alpha was 0.83.

#### Bulimic symptoms

We assessed the individual participant’s bulimic symptoms using the bulimia subscale of the Eating Disorder Inventory (EDI) [36]. The responses to the seven items were measured based on a six-point Likert scale with anchors ranging from 1 (never) to 6 (always). Examples of the items are “I eat when I am upset” and “I eat moderately in front of others and stuff myself when they’re gone”. The subscale has previously been shown to have good internal consistency in a group of college women (0.82) [37]. In the current sample, the Cronbach’s alpha was .87.

#### Patient Health Questionnaire 4 (PHQ-4)

The two depression items (the 2 other items operationalize general anxiety) from the PHQ-4 were used to operationalize the participants’ depressive symptoms [38]. Participants indicated on a four-point Likert scale with anchors ranging from 0 (not at all) to 3 (nearly every day) how often over the past 2 weeks they had been bothered by the following problems: “Little interest or pleasure in doing things” and “Feeling down, depressed or hopeless”. The current study found a Pearson correlation of 0.62 between these two items.

#### Procedure

The study was first approved by the Ethics Committee of the Department of Psychology at the University of Groningen. Female students were approached as they entered the university library. After providing informed consent, the participants were asked to complete a questionnaire containing demographic information (i.e., height, weight and age), measures of body shame, bulimic symptoms, depressive symptomatology and negative urgency. They then submitted the questionnaire to a sealed container situated in the library building.

### Statistical analysis

The predicted moderated-mediation model was examined using the PROCESS macro for SPSS (version 23) which utilizes a bootstrap approach to model testing. The bootstrap approach is robust to the possible influences of non-normal samples [39–42]. In this regard, bootstrapping is a type of resampling where large numbers of smaller samples of the same size are repeatedly drawn, with replacement, from a single original sample often 1000s of times. This generates an empirically derived representation of the sampling distribution which is used for the construction of confidence intervals. Unlike the normal theory approach, no assumptions are made about the shape of the sampling distribution. As a result, bootstrap confidence intervals, using simulation studies, have been found to better respect the irregularity of sampling distributions and as a result yield inferences that are more likely to be accurate than when the normal theory approach is used [39–42]. When used to test hypotheses, the result is a test with higher power.

Following Hayes [39], the hypothesized indirect effect (model 4 [39]) and the moderation effect (model 1 [39]) were first examined independently. A moderated-mediation analysis (model 14 [39]) that estimated all parameters simultaneously was then carried out. This analysis provided an index of moderated mediation (i.e., the slope of the line representing the association between the moderator effect and the indirect effect), as well as estimates of the indirect effect and associated confidence intervals on specified levels of the moderator (i.e., − 1SD, mean, + 1SD). Each analysis utilized 5000 bootstrap re-samples, and significance was determined based on 95% bias-corrected confidence intervals. The models tested included BMI as the predictor variable, negative urgency as the moderator, body shame as the mediator and bulimic symptoms as the criterion variable. Depression was the only covariate and was included in each analysis.

## Results

### Descriptive and correlation analysis

Descriptive statistics and zero-order correlations were calculated as presented in Table 1

**Table 1:** Pearson correlations, means and standard deviations of the measured variables

	1.	2.	3.	4.	5.
1. BMI	–				
2. Body shame	0.222*	–			
3. Bulimic symptoms	0.308*	0.602*	–		
4. Negative urgency	0.046	0.493*	0.490*	–	
5. Depressive symptoms	0.040	0.385*	0.354*	0.534*	–
Mean	22.065	26.732	14.923	8.866	1.723
SD	3.320	8.694	5.837	2.812	1.467

The unstandardized Pearson correlation coefficients are reported for each variable

*BMI* body mass index

*p < 0.01

### Mediation analysis

Following Hayes [39], a simple mediation analysis examined the indirect effect of BMI on bulimic symptoms through body shame while controlling for depressive symptoms. An overall significant model was found [*F*(3, 205) = 48.392, *p *< 0.001], with 41.46% of the variance in bulimic symptoms explained by the predictor variables. There were also significant direct effects for BMI on body shame (*B* = 0.542, SE = 0.164, 95% CI [0.219, 0.866], *p* = 0.001), for body shame on bulimic symptoms (*B *= 0.336, SE = 0.040, 95% CI [0.258, 0.415], *p* < 0.001) and a total effect for BMI on bulimic symptoms (*B* = 0.52, SE = 0.11, 95% CI [0.303, 0.732], *p* < 0.001). Depressive symptoms also had a direct effect on bulimic symptoms (*B *= 0.611, SE = 0.231, 95% CI [0.156, 1.065]). Furthermore, both the indirect (*B *= 0.182, SE = 0.064, 95% CI [0.077, 0.328]) and the direct effect of the BMI on bulimic symptoms (*B* = 0.335, SE = 0.097, 95% CI [0.145, 0.525]) were significant. A normal theory test for the indirect effect (*B* = 0.226, SE = 0.072, *z *= 3.119, *p* = 0.002) was also significant.

### Moderation analysis

**Fig. 2:**
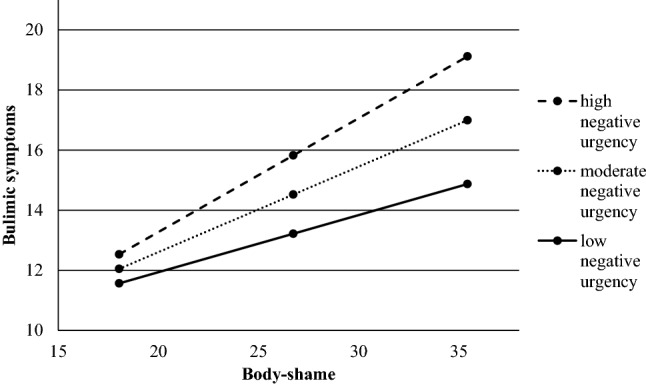
The interaction between body shame and low, average and high levels of negative urgency and their relationship with bulimic symptoms. The values are presented using unstandardized data

The second analysis examined the predicted moderating influence of negative urgency on the relationship between body shame and bulimic symptoms while controlling for depression symptoms. The overall model was significant (*F*(4, 204) = 39.700, *p *< 0.001), with 43.77% of the variance explained. While there were no significant direct effects, there was a significant interaction effect (*B* = 0.034, SE= 0.011, *t*(207) = 2.947, *p* = 0.004). Specifically, and as shown in Fig. 2, the relationship between body shame and bulimic symptoms increased in magnitude from low (− 1SD; *B *= 0.190, SE= 0.059, *p *< 0.002) to moderate (mean; *B *= 0.284, SE = 0.042, *p *< 0.001) to high (+ 1SD; *B *= 0.379, SE= 0.047, *p *< 0.001) with increasing levels of negative urgency.

### Moderated-mediation analysis

The index of moderated mediation [39], which reflects the slope of the line representing the association between moderator variable (negative urgency) and the indirect effect, was significant (*B *= 0.015, SE= 0.009, 95% CI [0.002, 0.036]). Thus, and consistent with our prediction, body shame mediated the relationship between BMI and bulimic symptoms across increasing levels of negative urgency. Further examination of the relationship between negative urgency and the indirect effect revealed that the conditional indirect effect of BMI on bulimic symptoms, through body shame, was significant at low (− 1SD; *B *= 0.096, SE= 0.050, 95% CI [0.023, 0.233]), moderate (Mean; *B *= 0.139, SE = 0.054, 95% CI [0.052, 0.268]) and high (+ 1SD; *B *= 0.182, SE= 0.066, 95% CI [0.068, 0.329]) levels of negative urgency.

## Discussion

In a sample of college women, it was predicted that body shame would mediate the relationship between body size and bulimic symptomatology. It was also predicted that negative urgency would exacerbate this mediation pathway, such that with increasing body shame and increasing negative urgency, the greater would be the bulimic symptomatology. Finally, it was expected that the predicted moderated-mediation relationships would occur over and above persistent levels of depression. All results were in line with predictions.

The significant moderated-mediation model reported in this study is, firstly, consistent with the contemporary sociocultural theory of women’s body image disturbance, whereby a woman’s negative thoughts and feelings about her body arise because she perceives that her body is discrepant from the cultural esthetic body standard [14]. Secondly, it converges with research suggesting that body size may influence disordered eating indirectly through its effects on negative affect [43]. Thirdly, the model is in line with research linking body shame to bulimic symptomatology [20, 22]. Fourthly, the moderating role of negative urgency is consistent with research not only suggesting negative urgency is the most predictive component of impulsivity for bulimia nervosa, but also with research and theory indicating that negative urgency influences bulimic symptomatology in the context of negative emotion [29, 30, 44]. Placing the significant moderated-mediation model within escape theory [23], our findings suggest that with increasing body size the young women in this sample felt more body shame at being too distant from the cultural esthetic standard and as a result engaged in more binging and purging to “escape” from such an aversive experience. Moreover, those women who were also higher in negative urgency, appear to be especially likely to engage in escape behaviors in the form of bulimic symptomatology when experiencing body shame specifically and not just when experiencing current depressive symptoms.

It is important to note here that the mean BMI of the sample was well within the normal range and suggests that many college women experience body shame even though they are not overweight. This supports contemporary sociocultural theory which purports that in modern Western society many women have internalized an unrealistic and unachievable thin ideal [15]. Presumably, comparison with this internal body standard leads to many women may experience body shame despite falling within a normal body range [14]. Such being the case, the findings of this study are also consistent with research indicating that women may experience bulimic symptomatology despite not being overweight [45].

Taken together, it would seem that women with both a higher body size and greater negative urgency may be particularly vulnerable to significant levels of bulimic symptomatology. Central to this vulnerability is that these women not only experience more body shame but unfortunately are also likely respond to this aversive emotional experience rashly in the form of behaviors symptomatic of bulimia nervosa. It could be, therefore, that focusing on the emotion of body shame rather than a general measure of body dissatisfaction may have greater potential clinical utility for health professionals. In this regard, such a focus would provide greater insight into the phenomenology of the body image experience and, furthermore, interventions can be targeted at developing specific adaptive emotional regulation strategies [46]. However, it is important to recognize that the focus of this study was on body shame. Recent research on body dysmorphic phenomenology suggests that body shame and general shame differentially influence disordered eating, with the former influencing body-related thoughts and behaviors and the latter influencing wider psychosocial functioning [22, 47]. Accordingly, a potential avenue of future research should be to examine the independent roles of general shame and body shame in our predicted model.

Thus, for women higher in both body size and negative urgency, individual treatment should perhaps focus on addressing the recognition, intensity and tolerance of body shame experiences specifically. Such an intervention could involve dialectical behavior therapy which utilises mindfulness, distress tolerance and emotion regulation skills, and has been found to be effective in this regard [48, 49]. Indeed, research would suggest that a particular focus of the intervention would be to enhance emotion regulation in emotion-inducing situations. Within the body image domain, situations that involve body checking (behavior aimed at acquiring information about body size, shape and weight) tend to make salient a discrepancy from the thin ideal body standard and, by so doing, evoke negative emotions like body shame [50]. Under such circumstances, therapeutic interventions could help women with greater body size and negative urgency cope more effectively with the consequences of body checking by enhancing their decentering abilities to attenuate body shame as well as reduce their attempts to control or avoid the body shame experience [51]. Furthermore, given the role of fear of compassion in fueling body shame, an effective preventative and therapeutic intervention could also involve enhancing self-compassion in the context of being at variance from the cultural thin-ideal, as well as facilitate strategies to promote warm and supportive relationships with others [52, 53]. Finally, it is important to recognize that effective emotion regulation also involves focusing on actually preventing a negative emotional experience taking place [28]. Given the relationship between body size and body shame in this study, an important antecedent-focused emotion regulation strategy focused on preventing the generation of body shame would appear to be a useful. This could involve challenging the sociocultural meanings that women attach to thinness and fatness, critically evaluating manifestations of the sociocultural esthetic standard (i.e., the so called thin-ideal), as well as promoting a broader conception of beauty focused on the functioning of the body rather than bodily appearance [54].

Our findings should not be interpreted without consideration of several limitations. Firstly, this study incorporated a cross-sectional design. Such designs may often produce biased estimates of mediation because of assumptions about stationarity, stability and the equilibrium of variables [55]. In recognizing this limitation, the significant findings could still be said to have utility because they facilitate a preliminary understanding of how key predictor variables may interact to increase bulimic symptomatology [40]. Nevertheless, our findings need to be replicated in a prospective design to produce truly prognostic results. Secondly, this study utilized a sample of college women. Although this sample can be considered to be taken from a population at risk for the development of bulimia nervosa [1], it is important to recognize that our college sample may differ from a clinical sample in terms of symptomatology, body shame and negative urgency. It is also worth noting that recent research suggests that levels of bulimic symptoms among college men are not insignificant [1], and thus there is a need to examine if our model is also relevant for male college students. Finally, we did not measure ethnicity; our sample was taken from a predominantly white northern European university. In this regard, some previous research [6], using a college sample, has reported a role for impulsivity in exacerbating the effects of body shame on women’s bulimic symptomatology in their black participants and not in their white participants. It is essential to recognize that Higgins et al’s [6] findings are valuable because they shed light on a relatively understudied racial group with regard to eating disorder pathology. However, while cultural and methodological differences could underpin our divergent findings, the results of Higgins and colleagues do suggest that among white college students, the influence of body shame on bulimic symptomatology is not benign. Clearly, future research is required to confirm whether such differences do indeed exist between differing ethnicities.

The significant findings of our study suggest other interesting directions for future research. For example, body size was also directly related to bulimic symptomatology suggesting the presence of the other mediating mechanism. It could be that other discrepancy-related body emotions, such as envy or guilt, also play a role in energizing and directing bulimic symptomatology. Given the potential role of emotion regulation in the development and maintenance of disordered eating [48], future researchers should perhaps also begin to examine the potential role of specific regulatory strategies in explaining the influence of body emotions on pathology. For example, researchers could examine the relative impact of such strategies as rumination suppression and distraction, in mediating relationship between negative body emotion and disordered eating.

In sum, body shame was found to mediate the relationship between body size and bulimic symptomatology. This suggests that many women attempt to “escape” this negative emotional experience by engaging in the binging and purging behaviors that are characteristic of bulimia. Furthermore, women who were also higher in negative urgency were particularly likely to engage in such “escape” behaviors when experiencing body shame. Finally, it appears that it is body shame specifically, over and above any current depressive symptoms, which underpins our results. For women higher in both body size and negative urgency therapeutic interventions should focus on emotion-regulatory strategies that prevent or attenuate the development of body shame.

## Data Availability

The datasets generated and analyzed during the current study are not publicly available, but will be available from the corresponding author upon request.
